# Cortical Excitability Measured with nTMS and MEG during Stroke Recovery

**DOI:** 10.1155/2015/309546

**Published:** 2015-09-27

**Authors:** Jyrki P. Mäkelä, Pantelis Lioumis, Kristina Laaksonen, Nina Forss, Turgut Tatlisumak, Markku Kaste, Satu Mustanoja

**Affiliations:** ^1^BioMag Laboratory, HUS Medical Imaging Center, Helsinki University Hospital and University of Helsinki, 00029 Helsinki, Finland; ^2^Neuroscience Center, University of Helsinki, 00014 Helsinki, Finland; ^3^Department of Neurology, Helsinki University Hospital and University of Helsinki, 00029 Helsinki, Finland; ^4^Department of Neuroscience and Biomedical Engineering, Aalto University School of Science, P.O. Box 15100, 00076 Espoo, Finland; ^5^Institute of Neuroscience and Physiology, Sahlgrenska Academy, University of Gothenburg, 41345 Gothenburg, Sweden; ^6^Department of Neurology, Sahlgrenska University Hospital, 41345 Gothenburg, Sweden

## Abstract

*Objective*. Stroke alters cortical excitability both in the lesioned and in the nonlesioned hemisphere. Stroke recovery has been studied using transcranial magnetic stimulation (TMS). Spontaneous brain oscillations and somatosensory evoked fields (SEFs) measured by magnetoencephalography (MEG) are modified in stroke patients during recovery. *Methods*. We recorded SEFs and spontaneous MEG activity and motor threshold (MT) short intracortical inhibition (SICI) and intracortical facilitation (ICF) with navigated TMS (nTMS) at one and three months after first-ever hemispheric ischemic strokes. Changes of MEG and nTMS parameters attributed to gamma-aminobutyrate and glutamate transmission were compared. *Results*. ICF correlated with the strength and extent of SEF source areas depicted by MEG at three months. The nTMS MT and event-related desynchronization (ERD) of beta-band MEG activity and SICI and the beta-band MEG event-related synchronization (ERS) were correlated, but less strongly. *Conclusions*. This first report using sequential nTMS and MEG in stroke recovery found intra- and interhemispheric correlations of nTMS and MEG estimates of cortical excitability. ICF and SEF parameters, MT and the ERD of the lesioned hemisphere, and SICI and ERS of the nonlesioned hemisphere were correlated. Covarying excitability in the lesioned and nonlesioned hemispheres emphasizes the importance of the hemispheric balance of the excitability of the sensorimotor system.

## 1. Introduction

The motor system is a dynamic network of cortical and subcortical areas interacting through excitatory and inhibitory circuits, modulated by somatosensory input. The network balance is disturbed after stroke [1–3]. Modifications of cortical excitability enable recovery and reorganization of the remaining motor areas both in animal models [4, 5] and in humans [1, 6]. Transcranial magnetic stimulation (TMS) and magnetoencephalography (MEG) have both been applied in stroke patients to reveal cortical excitability changes.

Motor threshold (MT), that is, the minimal TMS intensity eliciting motor evoked potentials (MEPs), is related to membrane excitability as it is influenced by drugs affecting neuronal ion channels. Paired pulse TMS (PP-TMS) reveals short-interval intracortical inhibition (SICI), related to the activation of GABA-A receptors and intracortical facilitation (ICF) attributed mainly to glutamatergic NMDA receptor activation (for references, see [7]). In acute stroke, the MT is increased in the lesioned hemisphere (LH) whereas in the nonlesioned (NH) hemisphere it is normal [8] or decreased one month after stroke [9]. SICI is decreased in both hemispheres early after stroke; ICF is stronger in NH in severe than mild strokes [1, 6].

Finger movements and median nerve [10] or finger stimulation [11] modify spontaneous MEG oscillations over the sensorimotor cortex in the beta band (15–25 Hz). They are suppressed at 100–300 ms after tactile stimulation (event-related desynchronization; ERD), reflecting increased excitability, and increased at 500–1000 ms (event-related synchronization; ERS), reflecting removal of excitation [12, 13] or reduced excitability [14]. Inhibitory GABA-A agonist diazepam increases MEG beta activity [15, 16]. Combined MEG and magnetic resonance spectroscopy showed a linear relationship between the beta ERS strength and GABA concentration [17]. Beta ERS has been shown to be significantly weaker in the LH than NH; stronger ERS amplitude was correlated with a better affected hand function up to 3 months after stroke [18].* Reduction* of beta ERS, however, correlated with clinical improvement after physiotherapy of patients with chronic stroke [19]. Movement-related beta ERD was significantly reduced in LH of stroke patients [20].

The hand representation in the somatosensory cortex (S1), estimated by somatosensory evoked fields (SEFs), is the largest one month after stroke and its increase was correlated with the affected hand function [21]. In TMS mapping, hand motor representation expands in the LH up to 1 month [22]. In animal models, somatosensory reorganization is activated immediately after the lesion by diminished GABA-A-related inhibition and by glutamatergic activation after one month [5].

We recorded both nTMS and MEG from patients during stroke recovery. We hypothesized to see correlations between ERD and MT related to the early cortical excitability and between ERS and SICI, both attributed to GABA-A-related processes. Moreover, we expected that ICF, reflecting glutamatergic activity, would correlate with the somatosensory modifications in MEG, as glutamate is associated with late somatosensory plasticity [5].

## 2. Methods

MEG and nTMS were recorded from thirteen patients (age 67.3 ± 11 years, 8 women), with first-ever ischemic stroke in the middle cerebral artery territory one (T1) and three months after stroke (T2). Hand function was impaired in all patients. Six patients had a subcortical and seven patients had a subcorticocortical or cortical stroke (Table 1). At T1, one patient used amitriptyline 10 mg/day and another used citalopram 10 mg/day. One patient used zopiclone 7.5 mg for occasional sleeplessness. At T2, citalopram 10 mg/day was used by one patient. Other patients did not receive drugs known to modify cortical excitability. The ethical committee of the Hospital District of Helsinki and Uusimaa approved the project. Data from these patients has been presented previously [18, 21, 23, 24]. As the patients having both TMS and MEG recordings are a subset of the previous patient groups, we recalculated the group-level electrophysiological parameters to show that the present patient group is sufficiently similar to those reported previously (see Supplementary Tables 1 and 2 in Supplementary Material available online at http://dx.doi.org/10.1155/2015/309546). Only the features needed for interpretation of the present results are described.

### 2.1. nTMS Measurements

An eXimia navigated magnetic stimulator with a coplanar figure-of-eight coil of 70 mm wing radius (Nexstim Ltd., Helsinki, Finland) was used for nTMS. Patients' MRIs, required for navigation, were scanned at T1 and used also at T2.

The site eliciting the largest MEP amplitude in first dorsal interosseous muscle was located. The resting MT was determined from this site as described in [25]. The site was then stimulated by single TMS pulses at 110% of MT and by the PP-TMS at 90% for conditioning and 110% of MT for test pulses. Fifteen single pulses or pairs of conditioning and test pulses were delivered in each stimulation session. The interval between stimuli was 3.3 s and the intersession interval varied between 1 and 3 min. The ISI selected for the paired-pulse stimuli was 2 ms for SICI and 12 ms for ICF. The peak-to-peak amplitudes of MEPs elicited by PP-TMS were normalized by dividing them by the corresponding single-pulse MEP amplitude to simplify subject-to-subject comparisons [6].

### 2.2. MEG Recordings

MEG during rest and tactile stimulation of the thumb (D1), index (D2), and little finger (D5) were recorded with a 306-sensor neuromagnetometer (Elekta Neuromag, Helsinki, Finland) in BioMag laboratory, right before the nTMS measurement. The interfering signals were suppressed by Maxfilter software [26]. The signals were filtered through 0.03–308 Hz and digitized at 941 Hz.

Time-frequency representations (TFR [27]) in the 10–30 Hz band were calculated to define the frequency range of beta modulation, which was quantified by the temporal spectral evolution method (TSE [10]) from signals of 2 to 4 MEG sensors showing the strongest reactivity. Only the contralateral beta modifications (the affected hand stimulation for the LH and the unaffected hand for NH) were analyzed. Onset and offset of the ERD and ERS were defined as a time point when the signal differed 2 SDs from the baseline. The absolute ERD and ERS strengths were calculated from the peak amplitudes and converted into relative values in relation to the 300 ms prestimulus baseline [18].

For SEFs, about 120 responses were averaged for stimulation of D2 (ISI 3005 ms), and D1 and D5 (ISI 1005 ms) in separate sessions. The size of the hand representation in the SI was determined by calculating the Euclidean distance in *xyz*-space between the equivalent current dipoles (ECDs) of the earliest responses to D1 and D5 stimulation [21]. The amplitudes of SEFs to D2 stimulation [23] were used in the analyses.

SPSS 14.0 software was employed for statistical analysis (SPSS Inc., Chicago, Illinois, USA). Spearman's correlation coefficients were calculated between nTMS and MEG parameters. Multiple comparison correction was carried out according to the number of tests (*N* = 32) suggested by the four prior hypotheses (T1 and T2 were tested separately; LH and NH variations lead to four tests in each case; *N* = 4*∗*4*∗*2 = 32). The significance level was set at *p* < 0.05. We also present significances of correlation values without multiple comparisons and supply all correlation coefficients and corresponding *p* values (cf. [1, 9, 28] for a similar approach and [29] for its statistical aspects). The differences between nTMS and MEG values obtained at T1 and T2 were tested with Student's* t*-test.

## 3. Results

### 3.1. Navigated TMS

In the LH, MEPs were found in 11 patients both at T1 and at T2 and were present in the NH in all patients. Group average MTs did not change between measurements or hemispheres. Individual MT values are given in Supplementary Table 1. MT was higher for LH than NH in 9 patients at T1 (*p* < 0.05, binomial test) and in 10 patients at T2 (*p* < 0.05, binomial test). The MTs in the LH and NH correlated strongly both at T1 (*r* = .82, *p* < .01) and at T2 (*r* = .78, *p* < .01).

PP2ms stimulation of LH inhibited MEPs in 7 patients at both T1 and T2. PP2ms stimulations of NH did not inhibit MEPs (disinhibition; diminished SICI) in 5 patients at T1 and in 3 patients at T2. SICI values did not correlate significantly between the hemispheres at T1 or T2.

PP12ms stimulation of LH facilitated MEPs (ICF) in 10 out of 11 patients at T1 and in all patients at T2. In NH, ICF was induced in all patients at T1. At T2, ICF was not observed in 4 patients. MEP amplitudes elicited by PP12ms stimulations were correlated between the LH and NH at T1 (*r* = .62; *p* < .05) but not at T2 (Supplementary Table 2).

### 3.2. MEG

ERD started 140 ± 10 ms after tactile stimulation and peaked at 250 ± 10 ms. The subsequent ERS started at 520 ± 40 ms and peaked at 900 ± 70 ms. At T1, ERD was absent in one patient and ERS in two patients in the LH; ERS was missing from the NH in one patient. At T2, the ERD was present in every LH whereas ERS was absent in two. Both were present in all NHs. ERDs did not differ between the hemispheres. ERS was smaller in the LH than NH at T1 (46 ± 31% versus 63 ± 32%; *p* < .05); at T2, the difference was nonsignificant. SEFs from both hemispheres were detected in 12 patients at T1 and in all patients at T2. They were smaller in the LH than NH at T1 (25 nAm versus 32 nAm; *p* < .04) but not at T2. The SI hand representation area was larger in the LH than NH at T1 (12 ± 3 mm versus 10 ± 3 mm *p* < .003) but not at T2 (Supplementary Table 3).

### 3.3. Correlations between the nTMS and MEG

**Figure 1:**
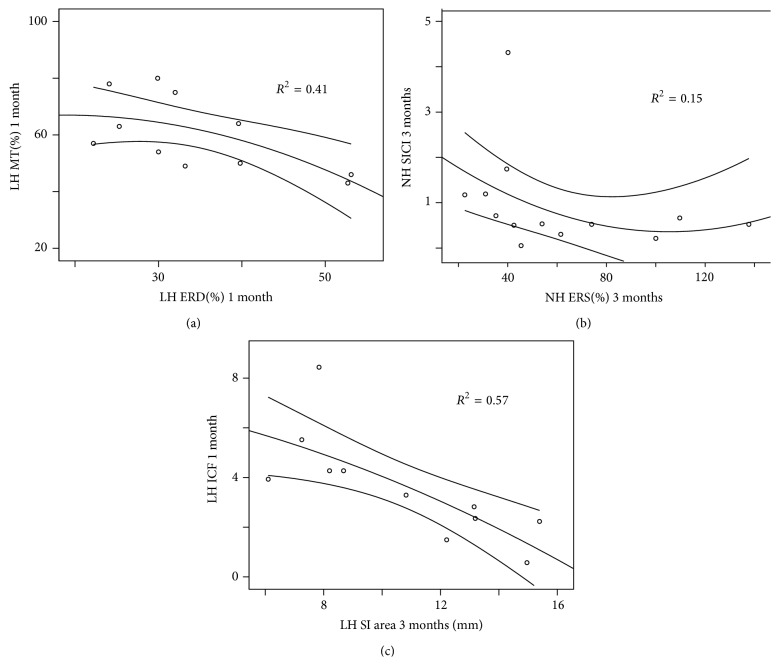
Scatterplots, quadratic fits, and 95% confidence intervals of the motor thresholds and ERD values in the lesioned hemisphere at 1 month (a), SICI and ERS values in the nonlesioned hemisphere at 3 months (b), and ICF values at 1 month and SI area at 3 months in the lesioned hemisphere (c).

We tested the correlations indicated by the selected hypotheses. The plots of the most relevant correlations are depicted in Figure 1 to show that they were not driven by outliers. All correlations are displayed in Table 2 to enable evaluation of significance of our hypotheses against general effects of the lesions.

*The MT and ERD* were correlated in the LH at T1 (*r* = −.66, *p* < .03), indicating that small ERD was associated with a high MT (Figure 1(a)). At T2, this correlation was weaker (*r* = −.58, *p* < .06). However, the MT of the LH correlated with the ERD of NH (*r* = −.62, *p* < .04), and the MT of the NH correlated with ERD of the LH (*r* = −.65, *p* < .02), suggesting that high MT was associated with a small ERD in the opposite hemisphere at T2 (Table 2).

*SICI and the ERS* did not correlate at T1 or in LH at T2. In the NH, high ERS was associated with a strong SICI (*r* = −.59, *p* < .04; Figure 1(b)). In addition to hypothesized correlations, SICI of the NH and ERD of the LH at T2 were correlated (*r* = −.82, *p* < .001), indicating that strong ERD in the LH was associated with a strong SICI in the NH. SICI in the LH was correlated also with the SI amplitude of the LH (*r* = −.64, *p* < .04), indicating that small SI amplitude was associated with a weak SICI (Table 2).

*ICF and SEF* parameters did not correlate at T1. At T2, ICF in the LH correlated with the S1 amplitude of LH (*r* = −.65, *p* < .03); if ICF was strong, the SI amplitude was small. In addition, ICF in the NH correlated (*r* = −.82, *p* < .001) with the SI finger representation area of the LH; this correlation remained significant also after Bonferroni correction (Table 2). Moreover, ICF in the LH at T1 was correlated (*r* = −.83; *p* < .002) with the SI finger representation area of LH at T2; high ICF at T1 resulted in a small hand representation area at T2 (Figure 1(c)).

## 4. Discussion

Our study is the first to compare MEG and nTMS excitability parameters during stroke recovery. Navigated TMS, not applied previously in longitudinal studies of stroke patients, enabled the precise replication of the stimulus site between separate measurements, adding reliability to the follow-up. We found correlations between cortical excitability estimates derived from nTMS and MEG.

As expected, we found correlations between MT and ERD, but in only one of the four possible intrahemispheric and two out of four interhemispheric conditions. The SICI and beta ERS, both attributed to GABAergic mechanisms, were correlated in one of their four possible intrahemispheric conditions (in the NH at T2). Interhemispheric correlations of SICI were not limited to ERS. One reason for this may be that various GABA-A receptor subtypes contribute to SICI. Nonselective GABA-A receptor activators modify SICI whereas the GABA-A1 receptor specific zolpidem did not [7]. Nonselective GABA-A agonist zopiclone increased MEG beta activity whereas zolpidem suppressed beta activity in the vicinity of stroke lesion [30]. Moreover, in healthy subjects, diazepam increased MEG ERD but did not affect ERS when the increase of baseline beta activity was taken into account [16]. This nonspecificity could contribute to the correlations of SICI with the ERD and MT as well.

Correlations between nTMS parameters and MEG ERD/ERS were stronger at T2 than at T1. Analogously, most TMS intracortical excitability measures did not correlate with the hand function acutely but did so 3 months after stroke [1]. Recovery of sensorimotor fMRI activation to digit stimulation from 1 to 3 months was correlated with final motor function [31], emphasizing the importance of this time period for stroke recovery.

ICF correlated with SI hand area size at T2. As ICF is attributed mainly to glutaminergic mechanisms, glutamate may contribute to stroke-induced plasticity. Somewhat surprisingly, high ICF at T1 correlated with small SI hand area at T2 (Figure 1(c)); thus, the narrowing towards normal hand representation size may be supported by glutaminergic activity. ICF did not correlate with the MEG ERD/ERS, and the SI hand area and beta ERD/ERS were not correlated [18]; this suggests different mechanisms underlying SICI and ICF (see [7] for a detailed discussion).

**Figure 2:**
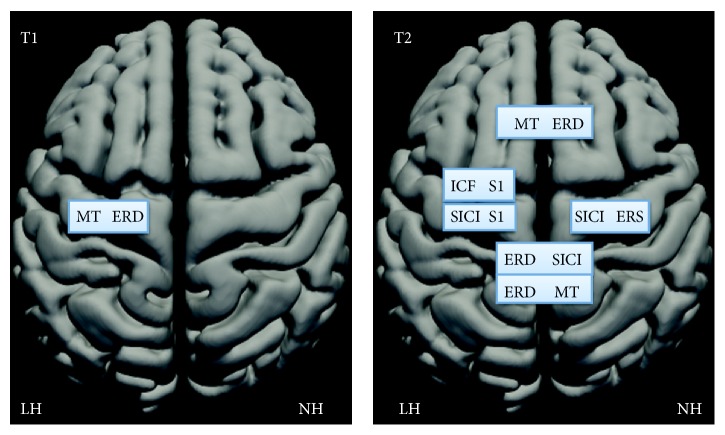
TMS and MEG parameters correlating at T1 (one month) and at T2 (three months), drawn on a schematic brain with lesioned (LH) and nonlesioned hemisphere (NH). The intrahemispheric connections are drawn on one hemisphere and interhemispheric connections on both hemispheres. Note strong increase of both intra- and interhemispheric correlations from one to three months after the stroke.

Several correlations emphasized interhemispheric connectivity (see Figure 2 and Table 2). For example, high MT was associated with a small ERD in the opposite hemisphere, and strong ERD in the LH was associated with a strong SICI in the NH. This suggests that the hemispheric balance of excitability is important in stroke recovery. Dexterity is impaired in both hands after unilateral subcortical middle cerebral artery stroke. Increased excitability within the unaffected motor cortex may cause imbalance between the homologous cortical motor areas and worsen also the ipsilesional hand coordination (for references, see [32]). MTs between the hemispheres were strongly correlated both at T1 and at T2. Thus, some functional correlations may relate to the modified general excitability properties of the motor system, instead of effects in the immediate vicinity of the stroke [28].

Correlations between MEG and TMS parameters of cortical excitability were relatively loose. Several factors may explain this feature. TMS results give direct information of the changes in the motor output and the immediate effects of TMS are relatively local. However, also subcortical and spinal processes affect the MEPs used to evaluate the TMS effects. MEG reveals the activity of the whole cortical mantle and enables mapping of network effects generated by stroke. MEG source analysis suggests mainly motor cortex origin of beta ERS [13, 33, 34]. However, in electrocorticography, recorded directly from the cortex, beta ERD and ERS appear outside of pre- and postcentral gyri [35], in supplementary motor cortex [36], or broadly from pre- and postcentral gyri, frontolateral and medial cortex [37, 38]. The widespread cortical generation of the ERD and ERS may make them resilient to small cortical strokes. Multitude of generators may contribute to considerable variability of source locations of beta ERD in stroke patients (cf. [20]). Multiple sources underlying MEG signals may also explain resilience of auditory evoked fields after small strokes [39]. Stronger correlations between ICF and SI parameters than between MT and SICI and ERS/ERD may, in part, result from spatially more limited source areas of S1 responses than those of ERD/ERS. It can be expected that MEG and nTMS produce complementary information about the effects of stroke on cortical networks. Moreover, MEG parameters in the* affected* hemisphere and nTMS indices in the* unaffected* hemisphere were correlated with the motor performance of the affected hand (cf. [18, 21, 24]). This emphasizes the importance of combining these two methods.

ERD in the 8–22 Hz band may reflect downregulation of intracortical inhibition in the human motor cortex, as TMS delivered during ERD is associated with increased MEP amplitudes and reduced SICI [40]. However, 1 Hz repetitive TMS over the motor cortex reduces MEPs to subsequent single TMS pulses, indicating inhibition, but* decreases* postmovement beta ERS [41], and intermittent theta burst TMS facilitates MEPs but* increases* postmovement beta ERS [42]. Beta ERS is reduced in patients with myoclonus epilepsy, indicating increased cortical excitability [43]. In line, SICI is decreased in myoclonus epilepsy patients; however, MT is* increased* [44, 45], and the silent period after the TMS pulse, reflecting motor cortical postsynaptic inhibition [7], is* prolonged,* indicating prevailing inhibitory cortical tonus [45]. Thus, only some aspects of the cortical excitability may be shared in excitability estimates obtained by TMS and MEG.

Our results suggest that some TMS and MEG excitability measures reflect the activity of the same transmitter systems. However, high MT and absence of ERD/ERS may also correlate because of severely affected sensorimotor connections between the periphery and the cortex. We detected SEFs in 12/13 patients and MEPs in 11/13 patients already at T1, indicating that both somatosensory and motor pathways were conveying signals. Motor function can be maintained despite significant damage to the corticospinal tract, as estimated from MT of stroke patients [1]. Moreover, we observed fewer correlations at T1, when the sensorimotor pathways probably were more affected, than at T2. Large lesions may create spurious correlations between the excitability parameters within LH. However, such spurious correlations should remain stable or decrease during recovery from T1 to T2 but not increase, as in our data.

The limitations of our study include the small size of the patient group as the precise features of structural and functional changes may differ among the patients. Cortical excitability is modified differently in cortical and subcortical strokes [46, 47]. This, however, should not alter our correlations between the TMS and MEG, as both were recorded from the same patients. Possible effects of medication on excitability should go in parallel for MEG and nTMS, as the patients were tested sequentially during the same day. The patients were not tested in the acute stage with TMS, and MEG recordings showed most dramatic ERD and ERS modifications between the acute phase and T1 [18]. Although MT in the LH is correlated with the paretic hand function in acute stroke, this correlation, however, weakens during recovery, and TMS intracortical excitability parameters correlated with the clinical performance best at 3 months [1]. Longer follow-up could have produced additional correlations. The 2 ms or 12 ms ISIs, selected for our paired-pulse stimuli, produce clear SICI and ICF in healthy subjects [48] and in stroke patients [28], but we did not test other parameters, which could have produced stronger correlations between nTMS and MEG.

## 5. Conclusions

ICF and SI response amplitude and area size, MT and the ERD of the hemisphere harboring the stroke lesion, and SICI and ERS of the nonlesioned hemisphere are correlated in stroke patients. Numerous correlations of the excitability parameters between the LH and NH emphasize the importance of the hemispheric balance of the excitatory-inhibitory properties of the sensorimotor system in analyzing the stroke-related dysfunction during stroke recovery.

## Supplementary Material

Supplementary material contains individual motor threshold values in both hemispheres of each patient at T1 (1 month) and T2 (three months) after the stroke (Table 1), mean values and standard deviations of the nTMS parameters at T1 and T2 (Table 2) and mean values and standard deviations of the MEG parameters at T1 and T2 (Table 3).

## Figures and Tables

**Table 1:** Patient demographics and behavioral scores.

Pt	Sex	Age	Les. site	Hemi	Les. class	Les. size	NIHSS1	NIHSS2	mRs1	mRs2	BI1	BI2	Sense loss
1	M	60	Hand MCx	R	C	0.1	0	0	2	1	100	100	−′
2	F	68	GP	L	SC	1	2	2	1	1	100	100	+′
3	F	72	Primary MCx	L	C	0.3	0	0	1	1	100	100	−′
4	F	46	BGp	L	C + SC	70	8	6	3	3	65	85	+′
5	M	60	MCA territory	L	C + SC	48	2	2	2	2	100	100	+′
6	F	85	Hand MCx	R	C	1	0	0	1	1	100	100	−′
7	F	69	EC + insular	L	SC	3	2	0	2	2	90	100	−′
8	M	70	MCA territory	R	C + SC	297	12	12	5	5	35	40	+′
9	M	62	EC	L	C + SC	5	1	1	3	2	85	100	−′
10	F	75	Caud	R	SC	5	2	1	3	3	91	100	−′
11	M	78	Caud	L	SC	10	2	1	2	1	100	100	+′
12	F	73	Thal	L	SC	3	1	1	2	1	100	100	+′
13	F	48	Thal	L	SC	1	1	1	2	2	100	100	−′
Mean	66.8					32	2.5	2	2.2	1.9	90.4	94.6	
SD	10.7					79	3.3	3.2	1	1.1	18.7	16.2	
Max	85					297	12	12	5	5	100	100	
Min	46					0.1	0	0	1	1	35	40	

Sex: M = male; F = female. Age (years). MCx = motor cortex damage, GB = globus pallidus, BGp = basal ganglia posterior part, MCA territory = extensive involvement of the middle cerebral artery territory, EC = external capsule, Caud = caudate, and Thal = thalamus. NIHSS score (maximum 42), Modified Rankin Scale (mRs) and Barthel Index (BI) at 1 and 3 months after stroke. Hemi = hemisphere affected by stroke. L = left. R = right. Lesion classification: C = cortical, S = subcortical, and C + SC = corticosubcortical lesion.

Lesion size (volume in mm^3^).

**Table 2:** Spearman correlations between the nTMS and MEG parameters at 1 month (1 mo) and three months (3 mo) after the stroke. The correlations between event-related desynchronization (ERD) and motor threshold (MT), event-related synchronization (ERS) and short-interval cortical inhibition (SICI), and intracortical facilitation (ICF) and somatosensory evoked field source strength (SI) and somatosensory hand representation area (SIhr), aligned with hypotheses, are depicted in bold font. ^*^Significance of *p* < .05; ^**^significance of *p* < .01 without multiple comparison correction; ^**^(marked with bold italic) statistical significance (*p* < .05) with multiple comparison correction (Bonferroni) for *N* = 32.

			1 mo							
			LH				NH			
			ERD	ERS	SI	SIhr	ERD	ERS	SI	SIhr
1 mo	LH	MT	**−.66** ^*^	−.06	−.36	−.21	**−.48**	−.53	−.41	−.27
		SICI	−.45	**−.05**	−.51	.42	−.22	**−.04**	−.28	.12
		ICF	−.39	.01	**−.22**	**−.10**	−.06	−.07	**.30**	**−.19**
	NH	MT	**−.43**	−.12	−.24	−.21	**−.14**	−.37	−.49	.02
		SICI	.17	**−.03**	−.22	.26	.08	**.08**	−.16	.26
		ICF	−.51	−.11	**.16**	**−.02**	−.18	−.19	**.44**	**−.06**

			3 mo							
			LH				NH			
			ERD	ERS	SI	SIhr	ERD	ERS	SI	SIhr

3 mo	LH	MT	**−.58**	.11	−.14	.06	**−.62** ^*^	−.19	.04	.13
		SICI	−.27	**.32**	−.64^∗;^	.46	.06	**.20**	−.25	−.01
		ICF	.15	−.21	**−.65** ^**^	**−.28**	.46	.42	**.20**	**−.17**
	NH	MT	**−.65** ^*^	−.26	−.50	−.03	**−.48**	−.26	−.41	−.15
		SICI	−.82^**^	**−.51**	−.44	−.13	−.62^*^	**−.59** ^*^	−.07	−.05
		ICF	−.30	.04	**−.22**	***−.82*** ^**^	.30	.27	**.10**	**−.12**
