# Supplementary material for: Cortical Excitability Measured with nTMS and MEG during Stroke Recovery
Source: Neural Plast. 2015 Sep 27;2015:309546. doi: 10.1155/2015/309546 (PMC4600492; doi:10.1155/2015/309546)
Supplement: Supplementary file 1 — Supplementary material contains individual motor threshold values in both hemispheres of each patient at T1 (1 month) and T2 (three months) after the stroke (Table 1), mean values and standard deviations of the nTMS parameters at T1 and T2 (Table 2) and mean values and standard deviations of the MEG parameters at T1 and T2 (Table 3). [file 309546.f1.pdf]

Supplementary Table 1: Individual motor thresholds as a percentage of maximum stimulator output from the lesioned (LH) and non-lesioned (NH) hemispheres at T1(1 month) and T2 (three months) after the stroke. S.D. indicates standard deviation.

Patient	LH T1	LH T2	NH T1	NH T2
1	n.d.	n.d	85	91
2	50	56	55	51
3	75	78	57	58
4	n.d	n.d	71	81
5	43	44	34	41
6	57	50	42	42
7	49	57	47	47
8	54	51	54	46
9	80	75	59	61
10	46	49	43	42
11	78	64	77	64
12	63	58	54	51
13	64	56	67	69
Mean ± S.D.	60±13	58±11	57±15	57±16

Supplementary Table 2: Mean values and standard deviations of the nTMS parameters at T1(1 month) and T2 (three months) after the stroke. MT indicates motor threshold as a percentage of the maximum stimulator output. Short intracortical inhibition (SICI) and intracortical facilitation (ICF) are given as normalized values; actual amplitude values have been divided with the MEP amplitude elicited by a single-pulse nTMS.

	Lesioned Hemisphere			Non-lesioned Hemisphere		
	MT	SICI	ICF	MT	SICI	ICF
T1	60±13%	0.8±0.6	3.6±2.1	57±15%	6.5±15.7	29.9±95.46
T2	58±11%	2.4±5.2	2.5±1.5	57±16%	1.0±1.1	2.4±3.0

Supplementary Table 3: Mean values and standard deviations of the MEG parameters at T1(1 month) and T2 (three months) after the stroke. Event-related desynchronization (ERD) and event-related synchronization (ERS) are given as a percentage of the prestimulus baseline amplitude in the selected frequency band. SI indicates the source strength of the somatosensory evoked fields elicited by stimulation of digit 2 (in nAm), and SIhr the distance of the representations of digit 1 and 5 in he hand somatosensory cortex (in mm). Values indicated with * differ significantly at p < .05, those indicated with ** differ at p < .003.

	Lesioned Hemisphere				Non-lesioned Hemisphere			
	ERD%	ERS%	SI (nAm)	SIhr (mm)	ERD%	ERS%	SI (nAm)	SIhr (mm)
T1	32±14	46.±31*	25 ±11*	12.±3**	35±10	63.±32*	32±1 3*	10±3**
T2	36±11	47±32	22 ±14	10.±3	37±13	61±35	23±22	10±3
